# A Sustainable Route for CF/PA6 Composite Waste: From Scrap to Solutions

**DOI:** 10.1021/acsomega.6c03897

**Published:** 2026-05-15

**Authors:** Larissa Stieven Montagna, Guilherme Ferreira de Melo Morgado, Luis Felipe de Paula Santos, Juliano Marini, Fabio Roberto Passador, Mirabel Cerqueira Rezende

**Affiliations:** † Polymer and Biopolymer Technology Laboratory (TecPBio), 28105 Federal University of São Paulo (UNIFESP), 330 Talim St., 12231-280 São José dos Campos, Brazil; ‡ Lightweight Structures Laboratory (LEL), Instituto de Pesquisas Tecnológicas do Estado de São Paulo (IPT), 550 Dr. Altino Bondensan St., 12230-002 São José dos Campos, Brazil; § Department of Materials Engineering, 67828 Federal University of São Carlos (UFSCar), Rod. Washington Luiz, km 235, 13565-905 São Carlos, Brazil

## Abstract

The recycling of carbon fiber reinforced thermoplastics (CFRTP) is intrinsically complex, as it encompasses multiple interdependent variables, including the quality and heterogeneity of the incoming waste stream, as well as limitations on end-use applications, often restricted to nonstructural components, governed by the resulting mechanical and physicochemical properties of the recycled composite. Therefore, this work aimed to propose a sustainable route for the mechanical recycling of primary carbon fiber (CF)/polyamide 6 (PA6) composite blanket scrap preconsumer waste originating from the processing of components in the automotive sector. The waste was cut into specific geometric shapes, such as squares and triangles of two sizes (small: ∼5 cm; large: ∼10 cm), and placed in an aluminum mold, followed by hot compression molding to produce four reprocessed laminates. The laminates were inspected by ultrasound, and the volume fractions of reinforcement, matrix, and porosity were determined using the acid digestion method. The reprocessed laminates were evaluated for their thermal properties (differential scanning calorimetry, DSC, and thermogravimetric analysis, TGA), mechanical properties (interlaminar shear strength, ILSS, flexural strength, and Izod impact strength), and the morphological characteristics of the fracture surface. The results indicated superior values in the ILSS test, and the fracture surface morphology revealed satisfactory positioning, good adhesion, and interaction between the matrix and reinforcement in the reprocessed laminates. However, the patch arrangement and, mainly, the random orientation of the CF resulted in a drastic reduction in flexural performance. Laminate C, with smaller, triangular patches, resulted in greater impact resistance because it acted as a barrier to crack propagation and energy absorption. Reprocessing thermoplastic composite scraps proved feasible and satisfactory. Highlights: The reuse of carbon fiber reinforced thermoplastic composite scraps can be a viable solution for obtaining a secondary product.

## Graphical abstract

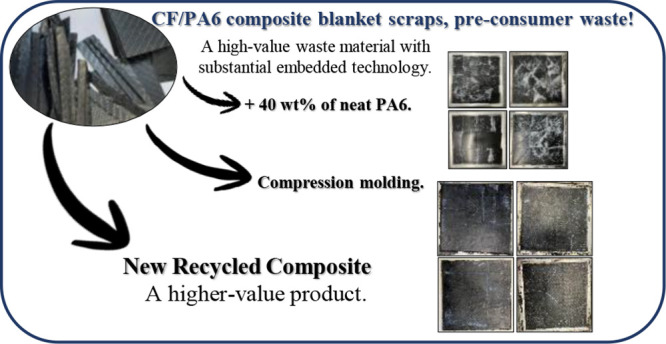

## Introduction

In recent decades, the growing demand for components and parts made from carbon fiber reinforced thermoplastics (CFRTP) has been driven by their excellent performance throughout their service life. CFRTP are widely employed in machinery, medical equipment, vehicles, aircraft, wind turbine blades, and sporting goods, as well as in many other applications across a wide range of industries.^1^ Along with this growing demand, the production of carbon fibers (CF) has also increased to meet the rising consumption of this reinforcement. Since their introduction in the 1960s, global demand for CF has risen by approximately 2000%. In the last three decades alone, it has increased from 6.5 kt in 1993 to 150 kt in 2023.^2^ Consequently, the use of high-performance thermoplastic matrices such as polyamide (PA), poly­(ether ether ketone) (PEEK), poly­(ether ketone ketone) (PEKK), poly­(ether imide) (PEI), poly­(phenylene sulfide) (PPS), poly­(aryl ether ketone) (PAEK), and others has also grown significantly.^3–5^

However, the high demand for CFRTP is already leading to environmental, social, and commercial challenges, since a large amount of waste is generated during production, operation, and at the end of service life. Currently, CFRTP waste is typically sent to incineration plants and/or controlled landfills,^6^ as these materials are designed to meet market demand rather than to be recovered or recycled. Nevertheless, CFRTP components are known to be fully recyclable, including both the polymer matrix and the reinforcement.^7,8^ The challenge lies in the fact that CFRTP waste is a complex system, requiring coordination among waste generators, collectors, and specialized recyclers, which makes the recycling process both difficult and costly.^9^ Furthermore, recyclability depends heavily on the quality of the waste, which can be compromised during production, use, and final disposal by contaminants such as paints, solvents, sealants, release agents (both liquid and solid), and metal fragments. Therefore, careful analysis of CFRTP waste quality is necessary to determine the most suitable decontamination and/or destination process, thereby minimizing costs and environmental impacts.

Environmentally sustainable and economically viable strategies include material reuse, reprocessing, and recycling pathways. However, there is a clear difference between reuse, reprocessing, and recycling.^10,11^ The term reuse consists of using the material again, without transformation, that is, it may only involve simple physical operations (cutting, shaping, repositioning). Reprocessing involves thermal processing methods, taking advantage of the thermoplastic nature of the matrix, and includes steps such as shredding, melting, and new processing (compression, injection, and/or extrusion), and reprocessing is within the context of recycling. The term recycling, on the other hand, is considered quite broad, as it involves any process that makes it possible to recover the material, that is, to recover the value of the waste, making it possible to transform this waste into a new material, product, or raw material.^12^

Recycling processes of CFRTP waste can be through chemical, thermal, or mechanical processes.^13^ Chemical and thermal recycling processes are primarily aimed at recovering CF. Both methods involve decomposition of the polymer matrix: in chemical recycling, solvents are used, while in thermal recycling, the matrix is degraded at high temperatures (between 400 and 700 °C) in an oxygen-free atmosphere, yielding CF and degraded matrix residues.^5,14,15^ Although these processes can produce well-preserved CF, the recovered fibers generally exhibit reduced mechanical properties, especially in tensile strength, when compared with virgin CF.^16^ However, this limitation does not prevent their use in other applications.^17–20^ Both recycling routes are associated with high costs and operational challenges: chemical routes involve large volumes of solvents, while thermal recycling requires high temperatures. These factors can make the processes economically unfeasible, resulting in recovered material with lower quality and higher cost than virgin CF.^21^

Another recycling route that has attracted increasing attention from both researchers and industry, particularly when preserving the integrity of the CF is not required, is mechanical recycling, widely known as the thermal reprocessing of waste. This process involves comminuting the waste, followed by particle size separation, after which the resulting material can be incorporated as reinforcement into polymeric matrices, whether thermoplastic or thermoset, since particle size and particle size distribution are critical parameters governing the mechanical performance of the resulting recycled composite.^22,23^ Mechanical recycling is generally recommended for producing lower-performance products, such as consumer goods and components in the mobility sector. Furthermore, this method requires less infrastructure and, consequently, involves lower processing costs.^24^

A viable, profitable, and sustainable alternative for CFRTP waste disposal is reuse and reprocessing. Industrial sectors that employ thermoplastic and thermoset composites, typically in the form of semipreg, prepreg, or blankets, discard a significant amount of scrap of various sizes and in excellent condition during the component production, which makes them suitable for reuse. When the scraps are based on thermoplastic and thermosets (uncured) matrices, they can be randomly arranged in molds and subsequently consolidated by hot compression molding.^25–27^ De Souza et al.^26^ reuse the waste of uncured composite prepreg scraps from the ply cutting manufacturing process in the aerospace industry. Through this work, he avoided the disposal of this waste, as well as the costs associated with its incineration and the loss of resin. This work motivated Montagna et al.^25^ to investigate the recycling of thermoplastic composites, such as carbon fiber/polyphenylene sulfide (CF/PPS), by placing semipreg waste into molds, followed by reprocessing via hot compression molding. According to the authors, the results were promising and may serve as a basis for the development of new products through recycling processes.

This study focused on reprocessing CF/PA6 composite blanket waste generated during the manufacturing of automotive components. Recycled laminates were produced by compression molding using 60 wt % composite waste and 40 wt % virgin PA6 matrix. The quality of the recycled composite laminates was evaluated through nondestructive inspection, determination of matrix, reinforcement, and porosity volume fractions, and analysis of thermal and mechanical properties. The objective of this study was to establish a technically and economically viable end-of-life management route, minimizing the need for additional specialized labor that could increase process costs, while ensuring an environmentally sustainable pathway for the full recovery of a secondary composite material. Furthermore, this approach is intended to foster engagement with potential partners committed to the development and implementation of efficient end-of-life collection strategies.

## Methodology

### Materials

The thermoplastic laminated composite waste used in this study is preconsumer waste (process waste) originating from the industrial processing of automotive parts. It is clean, free of external contaminants, and has not been previously exposed to environmental or chemical degradation. These preconsumer residues are carbon fiber reinforced polyamide 6 (CF/PA6) material, consisting of five plies in a 2 × 2 twill weave, with a composite density of 1.45 g/cm^3^, grammage of 415 g/m^2^, and a resin content (RC) of 40 wt %. The material corresponds to Cetex TC910, supplied by Toray (England). Neat PA6, trade name Aegis H8202NLB, was provided by AdvanSix (USA) and has a density of 1.13 g/cm^3^ with a melt flow index (MFI) of 9.8 g/10 min (235 °C, 1 kg).

### Processing of Recycled Composites

The CF/PA6 composite waste was selected and cut into smaller pieces in the form of small (∼5 cm) and large (∼10 cm) squares and triangles (Figure 1A). Laminates A and B were made with scraps in the shape of small and large squares, respectively, and laminates C and D were made with scraps in the shape of small and large triangles, respectively.

**Figure 1:**
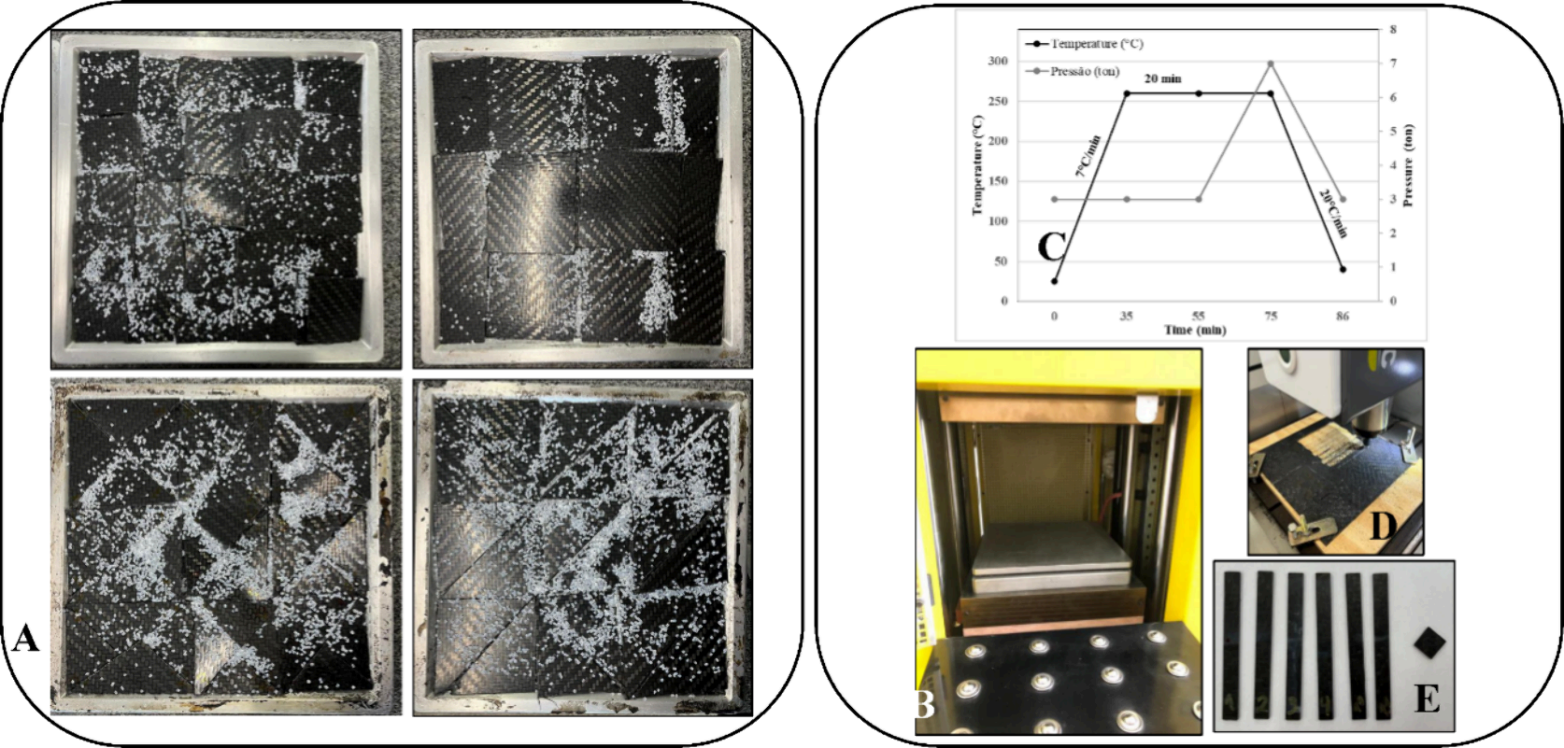
Processing methodology: (A) disposal of CF/PA6 waste with neat PA6 in the aluminum mold, (B) Luxor press, (C) processing parameters, (D) CNC for cutting, and (E) standardized specimens.

The CF/PA6 waste pieces and neat PA6 were dried in a vacuum oven (Solab SL-100) at 60 °C for 24 h. Four laminates with well-defined geometries (squares or triangles) were prepared. For each geometry, the pieces were positioned side by side with a random fiber orientation (Figure 1A). Neat PA6 (40 wt %) (white pellets shown in Figure 1A) was evenly distributed over the pieces to promote matrix infiltration into voids and ensure complete consolidation of the laminate. The reprocessed laminates were fabricated by hot compression using a Luxor press (LAB 40.15/1/ERA.AUT—OF 4272) (Figure 1B) following the methodology described by Dos Santos et al.^22^ The recycled composites were processed at 260 °C with a heating rate of 8 °C/min, under a minimum pressure of 3 bar. After reaching 260 °C, this condition was maintained for 15 min, followed by an additional 15 min under a constant pressure of 7 bar to ensure complete infiltration of the PA6 matrix into the CF tows. The laminates were cooled to 50 °C under a constant pressure of 7 bar, with a cooling rate of 7 °C/min (Figure 1C). Standardized specimens were subsequently cut using a CNC cutting machine (Router COBRA) equipped with a 3 mm quick cutter (Figure 1D,E).

### Determination of Volumetric Fractions by the Acid Digestion Method

The determination of the volumetric fractions of carbon fiber, polymer matrix, and void content was carried out in accordance with ASTM D3171 ^28^ and the methodology described by Montagna et al.^29^ The test was conducted in triplicate for each recycled sample. Composite waste specimens (15 mm × 15 mm × 2.5 mm) were dried in a furnace at 60 °C for 24 h to remove moisture. The dried specimens were then weighed and immersed in 80 mL of a concentrated sulfuric acid (H_2_SO_4_) solution (NEON, PA—Brazil) and heated to 150 °C on a hot plate (Corning, PC-420D) to promote the acid digestion of the polymer matrix. After 4 h, the beaker containing the acid solution was transferred to an ice bath, and 25 mL of hydrogen peroxide (H_2_O_2_) 30% (v/v) (Anidrol, 110 vol (30%), Brazil) was added to complete the digestion process, causing the CF to float on the surface of the solution (Figure 2).

**Figure 2:**
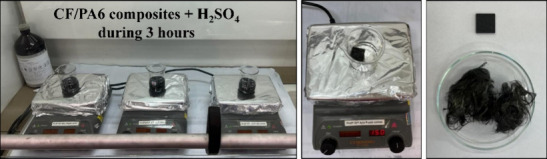
Schematic of the methodology used in the acid digestion process of recycled CF/PA6 composites.

The CF was repeatedly washed with distilled water to remove residual acid. They were then removed from the solution, dried in a furnace at 105 °C for 24 h, and cooled in a desiccator for 1 h. Finally, the CF mass was measured, and the volume fractions of CF (*V*_r_), matrix (*V*_m_), and void (*V*_v_) were calculated using eqs 1, 2, and 3, respectively.

$$V_{r}=\left(\frac{m_{f}}{m_{i}}\right)\times \frac{\rho_{c}}{\rho_{r}}\times 100$$ 1

$$V_{m}=\left(\frac{m_{i}-m_{f}}{m_{i}}\right)\times \frac{\rho_{c}}{\rho_{m}}\times 100$$ 2

$$V_{v}=100-(V_{r}+V_{m})$$ 3

Where *m*_i_ and *m*_f_ are the initial and final mass (g) of the CF/PA6 composites specimen and the CF resulting from the test, respectively; ρ_c_ is the density of composites (CF/PA6: 1.45 g/cm^3^), ρ_r_ is the density of the CF (1.76 g/cm^3^), and ρ_m_ is the density of PA6 (1.13 g/cm^3^).^24^

### Characterizations

The recycled composite laminates were inspected using a nondestructive ultrasound test (OmniScan SX) equipped with the OMNISX PA1664PR software, which converts the signals into images. The C-Scan method was employed with an operating voltage of 40 V, a frequency of 5 MHz, and step size of 0.077 mm.

Differential scanning calorimetry (DSC) analysis was performed using a TA Instruments Q2000 system, with nitrogen as the purge gas at a constant flow rate of 50 mL/min. Samples were heated from 25 to 250 °C at a rate of 10 °C/min. The degree of crystallinity (*X*_c_) was calculated using eq 4, where Δ*H*_m_ is the melting enthalpy, θ is the CF mass fraction in the composite, and Δ*H*_m_° is the melting enthalpy of a 100% crystalline PA6 polymer (240 J/g^30^).

$$X_{c}(\%)=\frac{\Delta H_{m}}{(1-\theta )\Delta H_{m}^{{}^{\circ}}}\times 100$$ 4

Thermogravimetric analysis (TGA) was performed using a TA Instruments Q50 analyzer with a heating rate of 20 °C/min, from 50 to 1000 °C, under a N_2_ atmosphere.

The Izod impact test was conducted according to ASTM D256 ^31^ using a CEAST/Instron impactor (model 9050) equipped with an 11 J hammer. A minimum of 5 unnotched specimens was tested.

The interlaminar shear strength (ILSS) test was carried out following ASTM D2344 ^32^ using an Instron universal testing machine (model 5982) equipped with a 100 kN load cell, operating at a crosshead speed of 1.0 mm/min. Five specimens (18 mm × 6 mm × 3 mm) of each laminate were evaluated. The interlaminar shear strength (τ_max_) was calculated by dividing the peak recorded reaction force (*P*) by the cross-sectional area (*b* × *h*), as shown in eq 5.

$$\tau_{\max}=0.75\frac{P}{\left(b\times h\right)}$$ 5

The three-point flexural strength test was performed in accordance with ASTM D7264/D7264M-21 ^33^ using an MTS testing machine (model 370.25) equipped with a 2.5 kN load cell and operated at a crosshead speed of 1 mm/min. Five specimens (125 mm × 13 mm × 3 mm) from each laminate were evaluated.

All mechanical test data were analyzed by one-way analysis of variance (ANOVA), followed by Tukey’s multiple comparisons test, using GraphPad Prism 6 software (GraphPad Software Inc., USA).

### Fractographic Analysis

Fracture surfaces from the ILSS tests were examined using optical microscopy (OM) and scanning electron microscopy (SEM). OM images were captured with a digital optical microscope (Instrutherm, model MP-150). SEM micrographs were obtained using an Inspect S50 microscope (FEI Company) equipped with secondary electron (SE) detectors, operated at an accelerating voltage of 20 kV and at different magnifications. Composite samples were placed in aluminum stubs with carbon tape and coated with a thin layer of gold for 120 s using a Quorum Q150RS Plus sputter coater.

## Results and Discussion

Nondestructive ultrasound inspection was employed to assess the structural integrity of the recycled laminates and to detect internal defects that could compromise the mechanical performance of these composites. Figure 3 shows the reprocessed laminates alongside their corresponding ultrasound images. The color scale on the right side of each image represents signal attenuation. Black or dark blue regions (0%) indicate high signal attenuation or areas with low reinforcement density. In contrast, red or orange regions (100%) correspond to full signal return, indicating defect-free areas with high reinforcement density.

**Figure 3:**
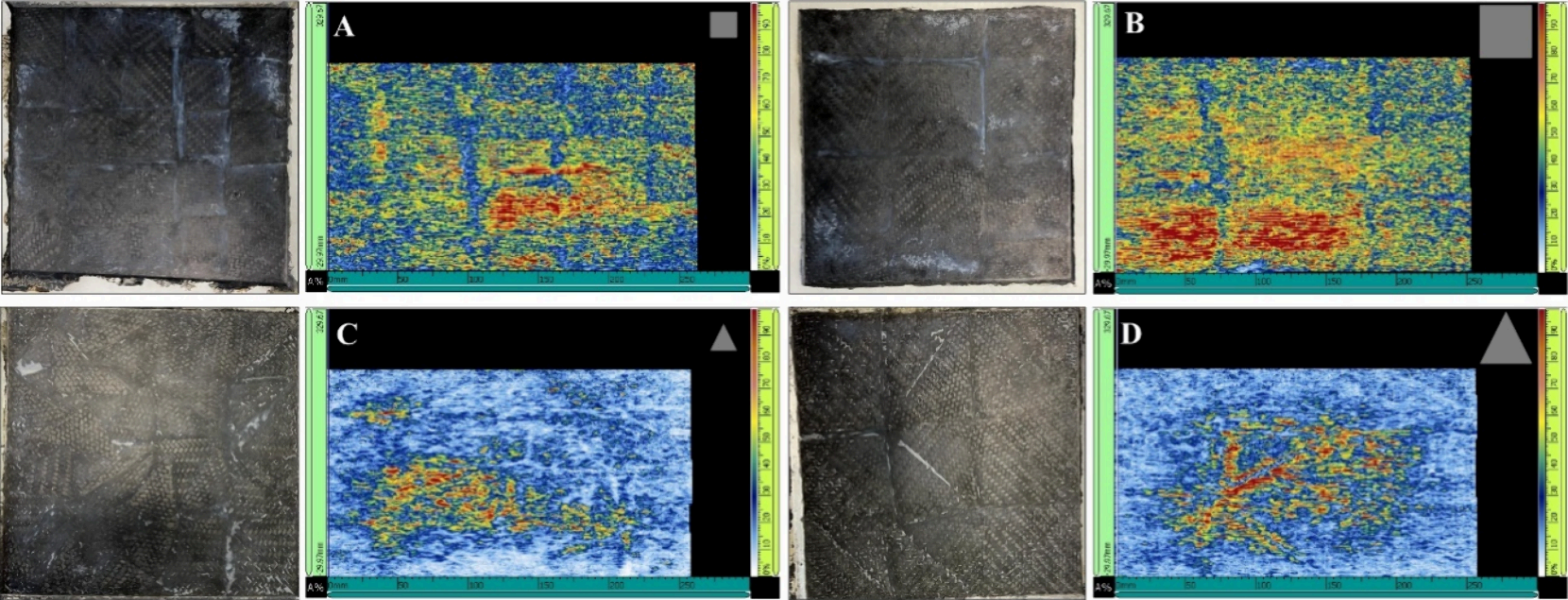
Reprocessed laminates with different geometries and dimensions of CF/PA6 composite scraps (A: small squares; B: large squares; C: small triangles; and D: larges triangles) and their respective ultrasound images.

In all laminate images, whitish regions correspond to pockets of neat polymer matrix added to facilitate processing. These regions appear as blue zones in the ultrasound scans, indicating low fiber density and revealing the joints between composite scraps.

Laminates A and B (Figure 3A,B), prepared with square scraps, exhibited very similar ultrasonic patterns. Reddish-orange areas, mainly in regions containing whole, seamless scraps, indicate zones with high fiber density. The various regions with high signal attenuation, represented by bluish-white areas, reveal zones with low reinforcement density; that is, they correspond to the presence of the polymeric matrix. This is consistent with the volumetric fraction of matrix and reinforcement, as the laminates contain a higher matrix content than reinforcement.

Laminates C and D (Figure 3C,D), prepared with scraps cut into small and large triangles, also presented whitish regions corresponding to the pure polymeric matrix, as observed for laminates A and B (Figure 3A,B). However, their ultrasonic images revealed more extensive areas with light bluish-white coloration, indicating higher signal attenuation and lower carbon fiber density. This behavior can be attributed to the triangular geometry of the scraps, as shorter lengths of the triangular carbon fibers reduce the continuity of the reinforcement.

Table 1 presents the volumetric fractions of PA6 matrix (*V*_m_), CF reinforcement (*V*_r_), and void content (*V*_v_) determined using the acid digestion method. According to the literature,^34^ CF/PA6 composites typically contain approximately 50 wt % (±10 wt %) of matrix and 50 wt % (±10 wt %) of CF. Montagna et al.^35^ reported volumetric fractions of CF/PA6 composites using the same acid digestion methodology, obtaining 51.3% CF, 48.4% polymer matrix, and 0.28% voids.

**Table 1:** Volumetric Fractions of CF, PA6 Matrix, and Voids in CF/PA6 Composites Obtained by Acid Digestion

	**volumetric content**		
**samples**	**voids (*V***_**v**_ **)**	**PA6 (*V***_**m**_ **)**	**carbon fiber (*V***_**r**_ **)**
A	4.0	57.9	38.1
B	3.6	57.3	39.0
C	3.9	57.7	38.4
D	4.1	58.1	37.8

The processed recycled composites exhibited average volumetric fractions of 57.8% (±0.3%) matrix, 38.3% (±0.5%) CF, and 3.9% (±0.1%) voids (Table 1). These values are higher than those reported by Montagna et al.,^35^ as 40 wt % of neat PA6 was added to the CF/PA6 composite waste, accounting for the increased polymer matrix fraction in the processed composites. Unlike composites with continuous fibers, the recycled composites contained scraps with irregular geometries and, consequently, variable lengths. During the arrangement of the composite residues in the mold with the neat polymer matrix, some regions may have lacked sufficient matrix, leading to void formation. Moreover, achieving uniform packing and adequate wetting of the CF by the thermoplastic matrix was challenging, and air entrapment and/or volatile release may also have occurred during processing, resulting in the formation of voids distributed throughout the recycled laminates.

The thermal properties of the recycled composites were analyzed to assess their stability and thermal integrity and to determine whether these thermal properties are preserved, ensuring reliable performance in future applications. Table 2 summarizes the DSC results of the reprocessed composites, while Figure 4 presents the DSC curves for the first and second heating cycles, as well as the cooling cycle. The DSC curves (Figure 4) exhibited similar behavior for all reprocessed laminates.

**Table 2:** DSC Results—Melting Temperature (*T*_m_), Melting Enthalpy (Δ*H*_m_), and Degree of Crystallinity (*X*_c_) in the First and Second Heating and Crystallization Temperature (*T*_c_) in the Cooling

	**1** **st** **heating**			**cooling**	**2** **nd** **heating**		
**samples**	***T***_**m**_ **(°C)**	**Δ*H***_**m**_ (J/g)	***X***_**c**_ **(%)**	***T***_**c**_ **(°C)**	***T***_**m**_ **(°C)**	**Δ*H***_**m**_ (J/g)	***X***_**c**_ **(%)**
A	222	29.3	19.7	189	221	29.7	20.0
B	222	30.5	20.8	189	221	32.0	21.9
C	222	39.4	26.7	189	222	34.1	23.1
D	222	34.4	23.1	189	222	29.0	19.4

**Figure 4:**
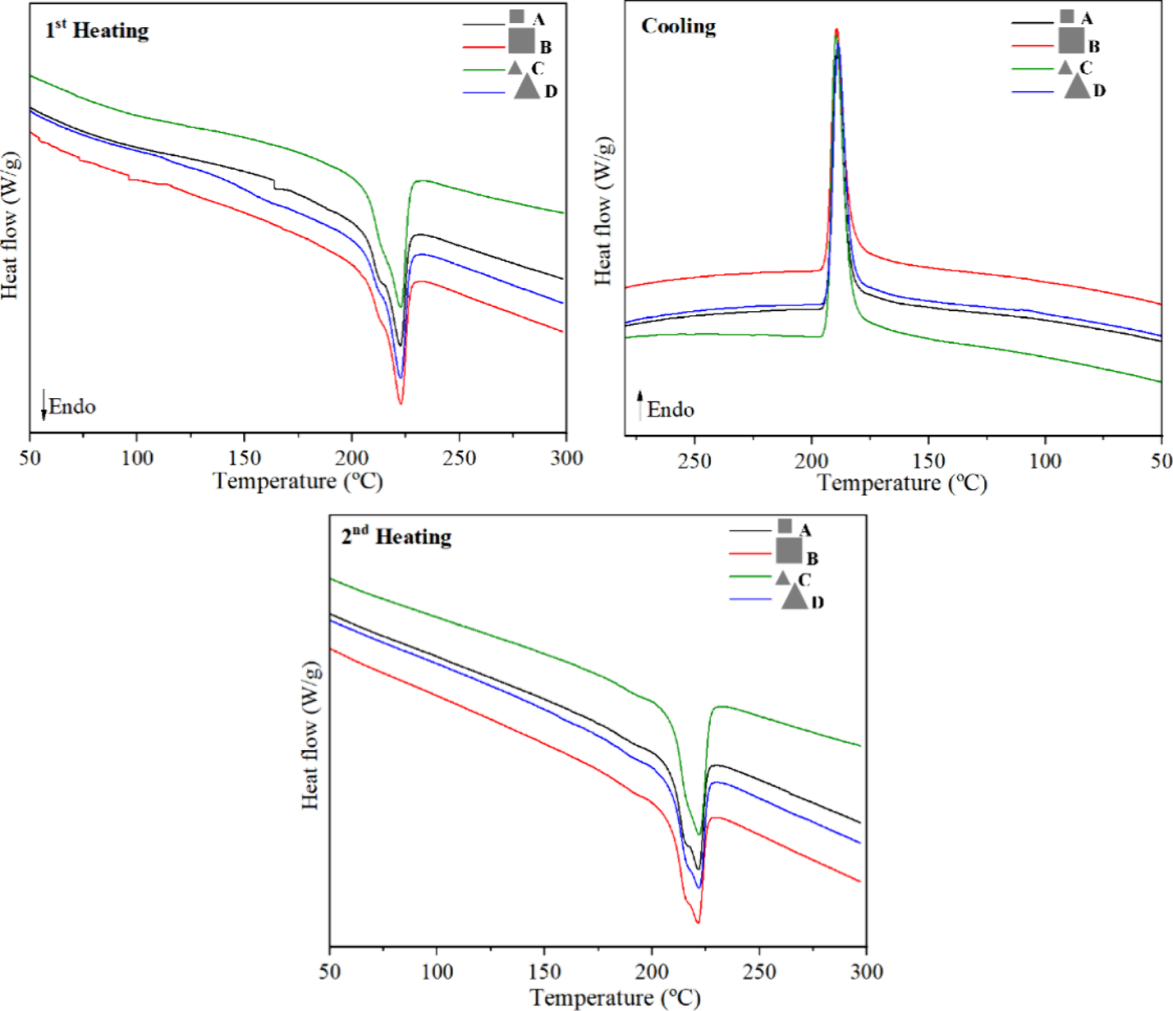
DSC curves of recycled laminates in the first and second heating and in the cooling of the recycled composite laminates.

All four reprocessed laminates exhibited the same melting temperatures (*T*_m_) of 222 °C in both the first and second heating cycles. In the study by Dos Santos et al.,^27^ the CF/PA6 composite and neat PA6 presented *T*_m_ values of 218 and 220 °C, respectively. The higher polymer matrix content in the reprocessed composites accounts for the observed *T*_m_ values, which are closer to that of the neat PA6 matrix, in agreement with the literature.^27^

Polymer reprocessing can alter the molar mass, especially for cases where processing occurs for long periods, as in this work, and consequently, affects the nucleation and growth of crystals, leading to a reduction in crystallization temperature (*T*_c_) due to imperfect and/or reduced crystal formation.^36^ All reprocessed laminates exhibited the same *T*_c_ of 189 °C, which is approximately 2% lower than the *T*_c_ of the CF/PA6 composite without the addition of neat PA6.^27^ This minor decrease in *T*_c_ suggests that the reprocessing of the composites did not significantly affect the final thermal properties of the recycled material.

Small differences were observed in the *X*_c_ values (Table 2) of the four processed laminates. Although the laminates were processed with the same parameters, the geometry and size of the composite scraps waste may have influenced the local flow behavior, packing efficiency, and fiber distribution during compression molding. These factors, in turn, affect polymer chain mobility and crystallization kinetics. According to the literature,^37^ CF/PA6 composites exhibit degrees of crystallinity (*X*_c_) of 22 and 19% in the first and second heating cycles, respectively. Laminate C (small triangular) showed the highest *X*_c_ values, 26.7 and 23.1% for the first and second heating cycles, which can be attributed to better packing and more efficient spatial rearrangement of the fragments during processing. The triangular geometry may have facilitated better accommodation within the mold, reducing voids and promoting more uniform heat and stress transfer. Furthermore, the greater number of edges and interfaces may act as heterogeneous nucleation sites, intensifying crystallization. Laminate A (small squares), despite having similar fiber lengths, may have resulted in less efficient packing and a higher probability of local restrictions on polymer chain mobility, limiting crystal growth and leading to a lower *X*_c_ (19.7 and 20%). Thus, the small variation in the degree of crystallinity values of the processed laminates was not governed solely by fiber length, but rather by a combination of geometric factors, interfacial area, and packing behavior.

Figure 5 shows the TGA curves, onset decomposition temperature (*T*_onset_), and residue content (%) of neat PA6, CF/PA6 composites waste, and the reprocessed laminates. The neat PA6 exhibits the highest *T*_onset_ value of 419 °C, as expected for a virgin polymer with no prior processing history. In the composites, the presence of CF influenced the decomposition behavior, as CF is thermally stable at high temperatures; therefore, the polymeric matrix can influence the *T*_onset_ of the composite. Similar behavior has been reported by De Oleveira et al.^22^ The CF/PA6 composite waste used to manufacture the reprocessed laminates had already undergone a prior processing cycle. Reprocessing further induces chain scission in the PA6 matrix, leading to a decrease in *T*_onset_ compared to the virgin composite.

**Figure 5:**
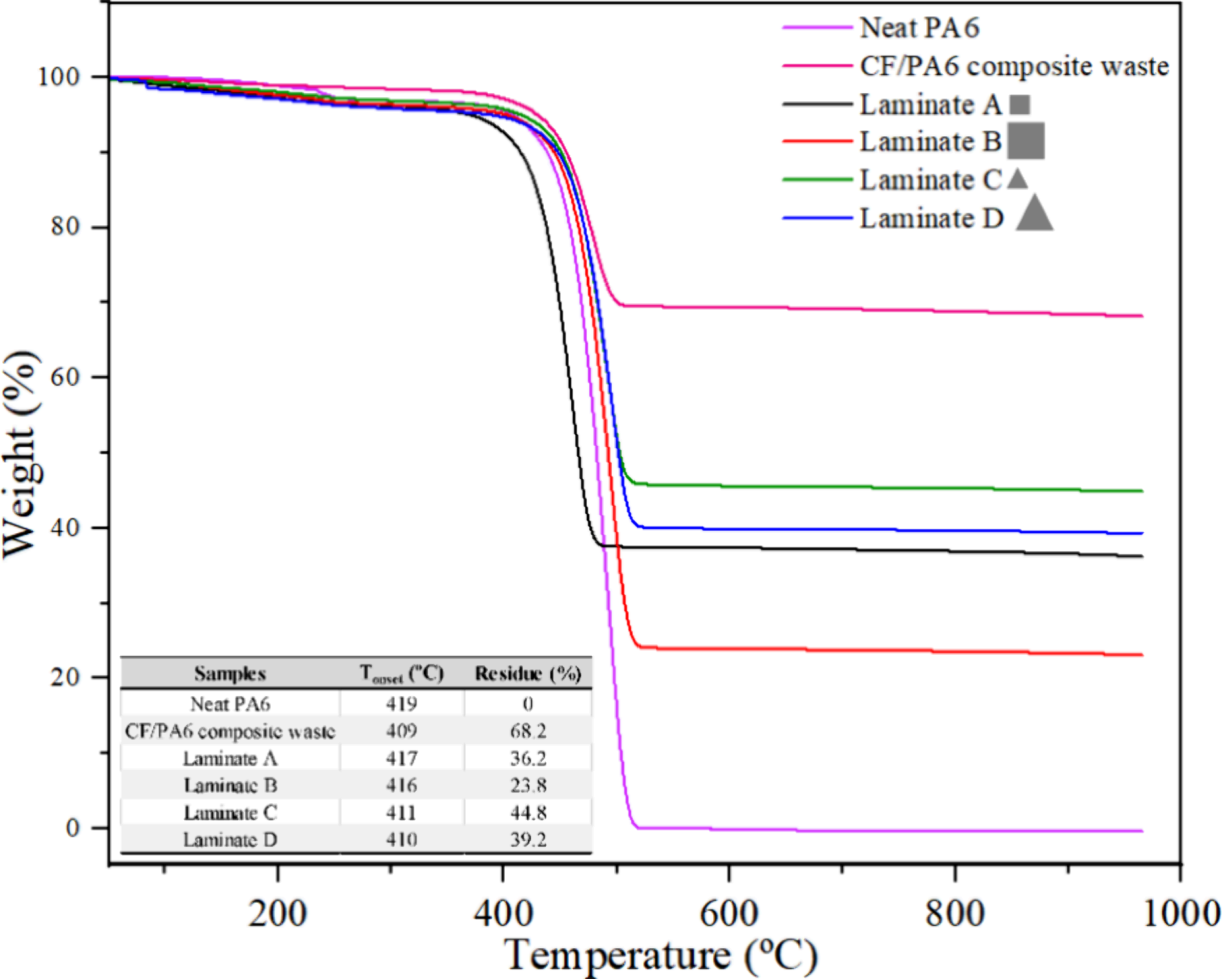
TGA curves, *T*_onset,_ and residue values of neat PA6, CF/PA6 composite waste, and reprocessed laminates with CF/PA6 composite waste.

An increase in the residue content of the reprocessed composites was expected, compared to the neat PA6, as the thermally stable CF does not decompose like the polymer matrix (in inert atmosphere), resulting in solid residue proportional to the CF content in the composite.

The reprocessed composites were evaluated using the ILSS test to assess adhesion and measure the shear strength between scrap layers, i.e., to evaluate the integrity of the matrix and the fiber-matrix interface. Figure 6 shows the ILSS values of the reprocessed composites and the reference sample (neat CF/PA6 composite), along with their respective optical microscopy images of the fracture surfaces illustrating the failure morphologies.

**Figure 6:**
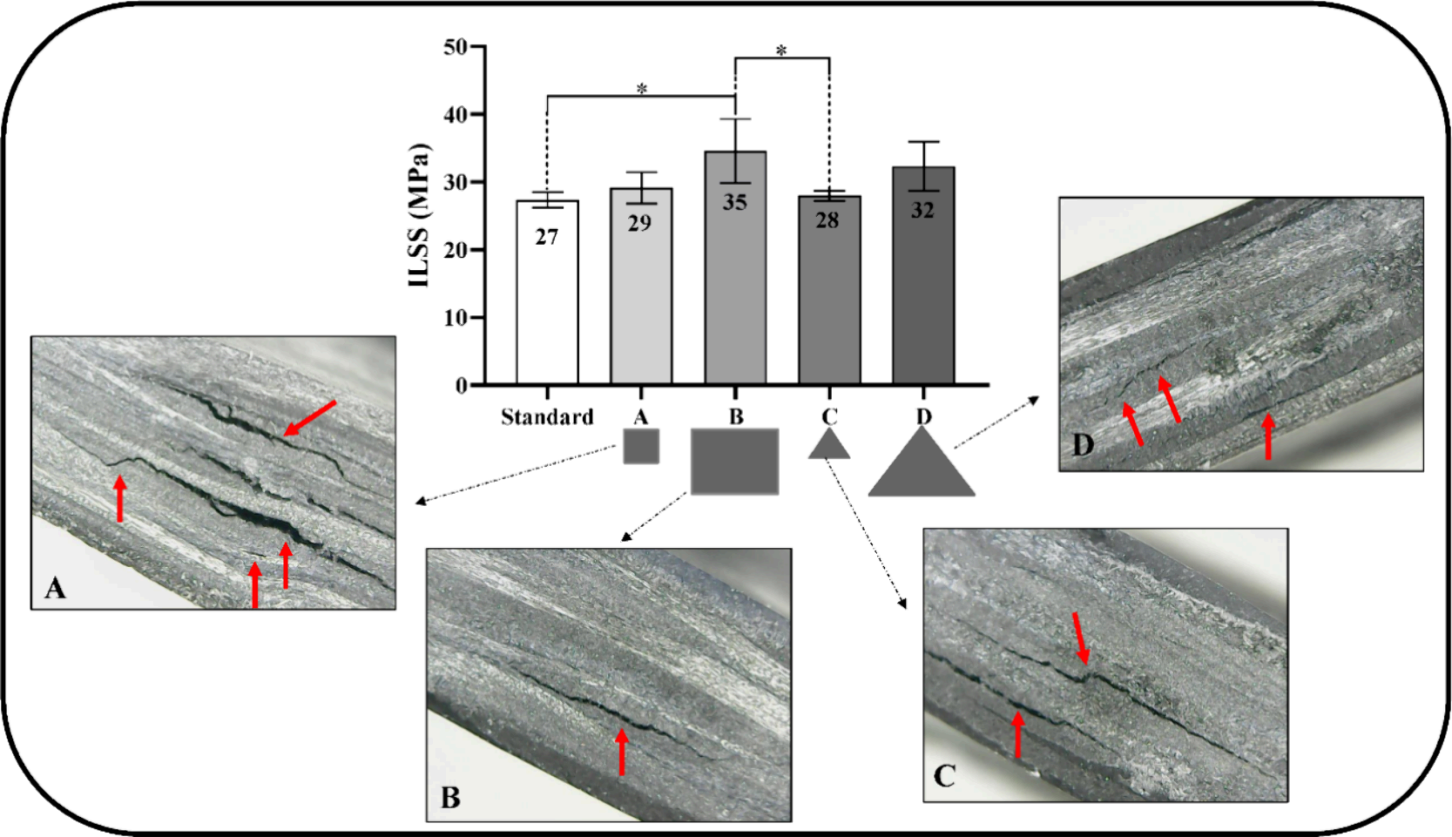
Interlaminar shear strength results and the representative images of the fracture of the ILSS test of the specimens: (A) small squares; (B) large squares; (C) small triangles; and (D) larges triangles. Results are given as mean ± standard deviation (SD) (*n* = 5). Asterisks (*) indicate statistical significance (*p ≤ 0.05).

Both reprocessed laminates exhibited higher ILSS values compared to the reference sample. The increase was particularly notable for laminates B (35 MPa) and D (32 MPa), corresponding to improvements of 23 and 16%, respectively, relative to the reference sample (27 MPa). Intralaminar fractures were observed in all samples, exhibiting similar behavior, which suggests good adhesion, integrity of the fiber-matrix interface, due to improved interfacial contact and tension transfer between adjacent reinforcements, indicating effective consolidation and adhesion. Fracture initiated in the matrix region parallel to the fibers and propagated preferentially through the matrix, halting at the opposite end of the CF region, as illustrated in Figure 6B–D. Laminates B and D were reprocessed with larger scraps (∼10 cm), squares (laminate B) and triangles (laminates D).

Laminates A (29 MPa) and C (28 MPa), which were reprocessed with smaller scraps (∼5 cm), exhibited lower ILSS values than laminates B and D, although their values remain close to that of the reference sample (27 MPa). Although it may seem counterintuitive, the improvement in ILSS values for laminates B and D can be attributed to differences in interlaminar architecture and stress transfer mechanisms. In the reference sample, ILSS is governed by the intrinsic fiber–matrix interface and relatively smooth, continuous laminar interfaces. In contrast, the reprocessed laminates exhibit a more complex interfacial structure due to the presence of multiple interfaces resulting from the arrangement of scraps between reinforcements, as well as increased surface roughness between adjacent layers. This leads to enhanced mechanical interlocking and frictional resistance at the interfaces, which may improve resistance to interlaminar shear loading.

Furthermore, during hot compression molding, localized resin flow and redistribution may have promoted improved wetting and consolidation at the interfaces between reinforcements, reducing brittle interlaminar regions and contributing to more effective stress transfer under shear loading.

The ILSS fracture images of Figure 6A–C show multiple cracks and fissures throughout the specimens. Intralaminar fracture was predominant in both samples, resulting from shear forces through the thickness induced by out-of-plane loading.^38^ The fracture morphologies further reveal the positioning and good adhesion of the CF/PA6 composite blanket patch layers, as well as the presence of longitudinal crack failures and intralaminar fractures (highlighted with red arrows), which are characteristic of this type of test.

Figure 7A presents the flexural strength values of reprocessed laminates with different geometries and patch sizes of CF/PA6 composite blankets. According to the Toray datasheet^29^ for the same material used in this work, the CF/PA6 composite has a flexural strength of 950 MPa (0°) following ASTM D790.^39^ It is worth noting that the flexural strength in the Toray datasheet refers to a controlled laminate with a defined fiber direction and a pattern different from the recycled laminates processed in this work. Therefore, the value used is only a general reference, and not a direct or equivalent reference. The reprocessed laminates showed a significant decrease in flexural strength. Several factors may contribute to this reduction, including the random arrangement of scraps in the mold, which leads to random fiber orientation and localized regions of low reinforcement concentration, thereby reducing fiber alignment in the load direction. Flexural strength is highly dependent on fiber orientation along the loading axis: short or poorly aligned fibers decrease stiffness and flexural performance.^40^ This behavior is likely present in the reprocessed laminates due to the random orientation of the CF/PA6 composite waste scraps.

**Figure 7:**
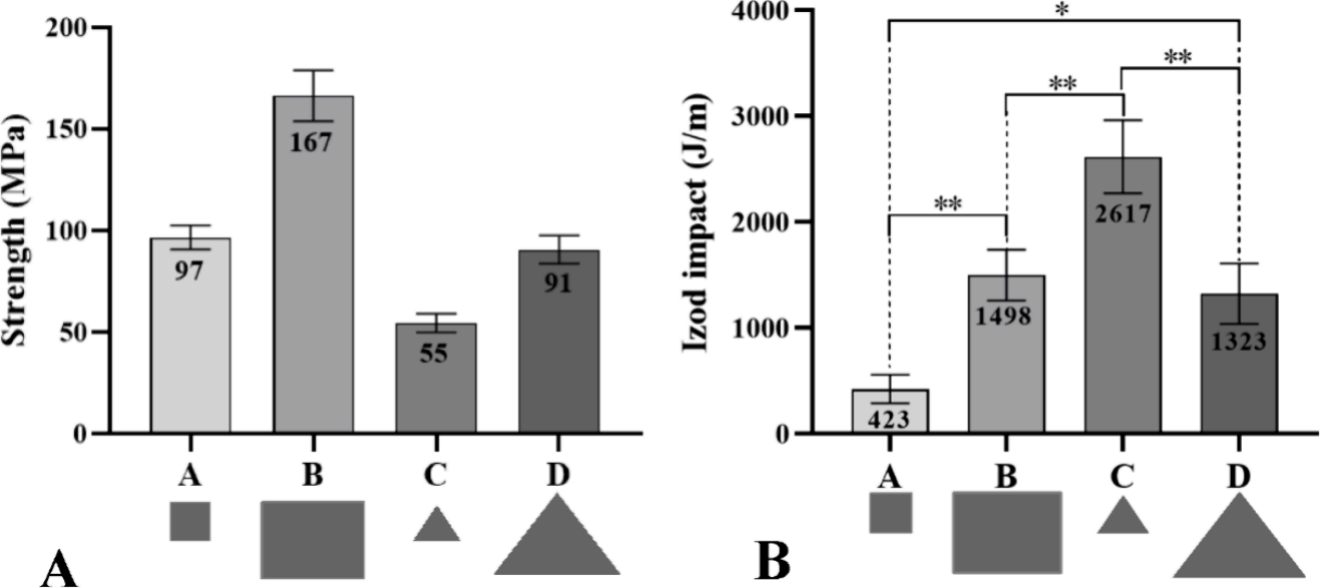
Mechanical test results: Three-point flexural strength values of the recycled composites laminates (A) and Izod impact test (B). Results are given as mean ± standard deviation (SD) (*n* = 5). Asterisks (*) indicate statistical significance (*p ≤ 0.05 and **p ≤ 0.01).

In the reprocessing of carbon fiber-reinforced polymer composites, it is important to consider that the surface treatment (sizing) of the CF may be damaged or removed, compromising adhesion between the fibers and the polymer matrix.^41^ Weak fiber-matrix adhesion hinders effective load transfer, negatively impacting flexural performance. However, micrographs (Figure 6) of the fracture surfaces from the ILSS test of the reprocessed laminates did not show regions with poor interfacial adhesion. Only intralaminar fractures were observed, which is characteristic of this type of composite test. Additionally, the presence of voids, porosity, and other defects can act as stress concentrators, promoting crack initiation and further reducing flexural strength.

Figure 7B shows the Izod impact strength results of the reprocessed laminates. In contrast to its poor flexural performance, laminate C (2617 J/m) exhibited the highest impact strength among all reprocessed laminates. The use of small triangular scraps in laminate C likely promoted the formation and random distribution of shorter CF within the recycled structure, which acted as barriers to crack propagation during impact loading. Furthermore, regarding Laminate C, the presence of smaller and/or more irregularly shaped spots promotes the deflection of cracks, branching, and energy dissipation mechanisms, thus increasing toughness. However, these same characteristics reduce structural integrity under static load, resulting in lower flexural strength. This microstructural configuration also favored matrix fragmentation into multiple matrix-fiber interfaces, CF displacement, delamination, and microcrack formation, mechanisms known to enhance energy absorption. These findings are consistent with the ultrasound images (Figure 3C), where the use of smaller triangular scraps resulted in short fibers and high signal attenuation, reflecting a lower fiber density and the presence of voids.

The other reprocessed laminates, A, B, and D, with different geometries and dimensions, exhibited Izod impact strength values 83, 43, and 49% lower, respectively, than laminate C. Two key factors may contribute to the formation of stress concentration zones, which facilitate crack propagation upon impact. The first is related to CF orientation: when the fibers are predominantly aligned in a single direction and the effect occurs out-of-plane, the composite is less able to dissipate energy, resulting in reduced impact strength. The second involves the nonuniform distribution of CF within the reprocessed laminate. Regions with fiber agglomeration or excess reinforcement tend to increase stiffness and brittleness. At the same time, matrix-rich areas create weak zones, both of which reduce the laminate′s ability to absorb impact energy.

Figure 8 shows SEM micrographs, at 1000× and 250× magnifications, of the ILSS fracture surfaces of the composite specimens reprocessed with CF/PA6 composite scraps of different geometries and dimensions. The specimens in Figure 8A–D were reprocessed with square-shaped scraps, where Figure 8A,B correspond to the smaller scraps (∼5 cm) and Figure 8C,D to larger scraps (∼10 cm). The higher-magnification images (Figure 8A,C (1000×) reveal fractured CF (highlighted with green arrows) well-adhered to the PA6 matrix, confirming strong fiber-matrix interfacial bonding. In Figure 8C, matrix-rich regions are evident (pink arrows), indicating localized areas of higher polymer concentration. The lower-magnification images (Figure 8B,D, 250×) display crack propagation paths associated with longitudinal intralaminar fracture (highlighted with blue arrows), with cracks initiating and terminating in a region containing CF oriented at 0°. In both lower and upper parts of the intralaminar fracture regions, fractured CF and small regions with accumulation in the polymer matrix are present.

**Figure 8:**
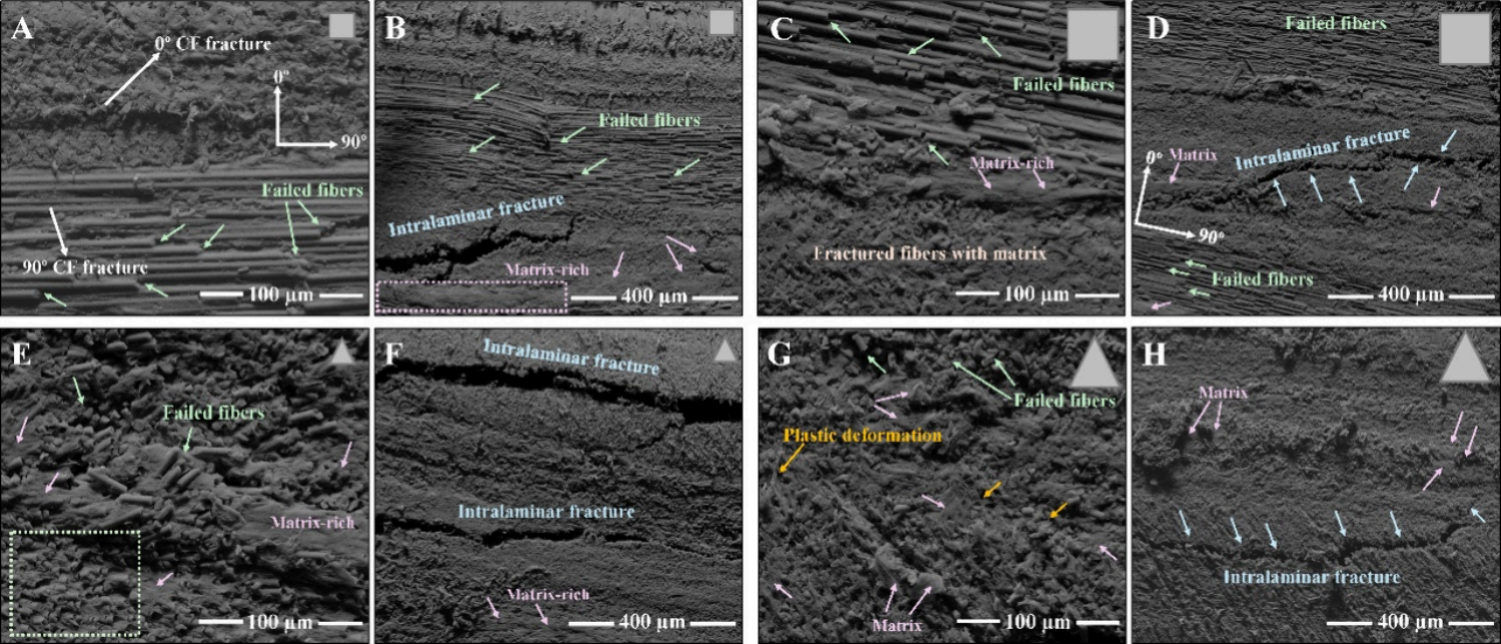
SEM micrographs with different magnifications (1000× and 250×) of reprocessed laminates with different geometries and dimensions of CF/PA6 composite scraps: Laminate A (A, 1000×, and B, 250×), Laminate B (C, 1000×, and D, 250×), Laminate C (E, 1000×, and F, 250×), and Laminate D (G, 1000×, and H, 250×).

Figure 8E,G (1000× magnification) show the ILSS fracture surfaces of laminates reprocessed using smaller (∼5 cm) and larger (∼10 cm) triangular-shaped scraps. Both micrographs show regions rich in polymer matrix (highlighted with pink arrows), and fractured CFs well-adhered to the polymer matrix are visible throughout the observed areas. In Figure 8G, localized plastic deformation is also evident in certain regions (orange arrows). The lower-magnification images (Figure 8F,H, 250×) display longitudinal intralaminar crack propagation (blue arrows), accompanied by small regions of matrix accumulation (highlighted with pink arrows) and fractured CFs well-adhered to the polymer matrix. Laminates reprocessed from CF/PA6 composite scraps with different geometries and dimensions exhibited the same intralaminar failure behavior, characteristic of ILSS tests on composites. Regions with polymer matrix accumulation are associated with a higher matrix than reinforcement content, as observed in the volume fraction results (Table 2).

## Conclusions

This study assessed the feasibility and demonstrated the reprocessing CF/PA6 composite blanket waste. Two waste geometries and sizes were selected to evaluate their influence on the final properties of the reprocessed composites.

The polymer matrix volume fraction in all reprocessed laminates was higher than the reference value due to the addition of 40 wt % neat PA6 to improve homogenization and reprocessing of the composites. Ultrasound images indicated regions with high signal attenuation, corresponding to low reinforcement density, i.e., matrix-rich regions, located at the patch joints. The reprocessing of CF/PA6 composite waste did not cause significant changes in the thermal stability of the laminates, with only a slight increase in the degree of crystallinity values.

The fracture surface morphology of the ILSS-tested specimens revealed intralaminar fractures, characteristic of this type of test, along with good adhesion and interaction between the matrix and reinforcement of the reprocessed laminates. This behavior corroborates the ILSS results, which were superior to the reference value, demonstrating that the positioning and adhesion of the CF/PA6 composite blanket scrap layers were satisfactory for this type of strength assessment.

In contrast, the flexural strength of the reprocessed laminates was approximately 90% lower than the reference value. This behavior may be linked to the arrangement of the scraps and the random orientation of the CF, which generated regions with low reinforcement concentration and poor CF alignment, resulting in a drastic reduction in flexural performance. The impact resistance of the laminate made with smaller triangular-shaped scraps (laminate C) was higher than that of the other laminates. This behavior is related to the reinforcement being smaller and acting as a barrier to crack propagation, in addition to acting as an energy absorption mechanism.

Therefore, about the mechanical properties evaluated for the four laminates processed from residues of different geometries and sizes, the apparent discrepancies arise from the intrinsic trade-off between stiffness/strength and toughness, as well as from the distinct mechanisms governing each test.
